# Properdin Deficiency Impairs Phagocytosis and Enhances Injury at Kidney Repair Phase Post Ischemia–Reperfusion

**DOI:** 10.3389/fimmu.2021.697760

**Published:** 2021-09-06

**Authors:** Yuanyuan Wu, Zinah D. Zwaini, Nigel J. Brunskill, Xinyue Zhang, Hui Wang, Ravinder Chana, Cordula M. Stover, Bin Yang

**Affiliations:** ^1^Department of Cardiovascular Sciences, College of Life Sciences, University of Leicester, University Hospitals of Leicester NHS Trust, Leicester, United Kingdom; ^2^Basic Medical Research Centre, Medical School of Nantong University, Nantong, China; ^3^Department of Respiratory Sciences, College of Life Sciences, University of Leicester, Leicester, United Kingdom; ^4^Nantong-Leicester Joint Institute of Kidney Science, Department of Nephrology, Affiliated Hospital of Nantong University, Nantong, China

**Keywords:** apoptosis, inflammation, ischemia–reperfusion injury, phagocytosis, properdin, repair

## Abstract

Properdin, a positive regulator of complement alternative pathway, participates in renal ischemia–reperfusion (IR) injury and also acts as a pattern-recognition molecule affecting apoptotic T-cell clearance. However, the role of properdin in tubular epithelial cells (TECs) at the repair phase post IR injury is not well defined. This study revealed that properdin knockout (P^KO^) mice exhibited greater injury in renal function and histology than wild-type (WT) mice post 72-h IR, with more apoptotic cells and macrophages in tubular lumina, increased active caspase-3 and HMGB1, but better histological structure at 24 h. Raised erythropoietin receptor by IR was furthered by P^KO^ and positively correlated with injury and repair markers. Properdin in WT kidneys was also upregulated by IR, while H_2_O_2_-increased properdin in TECs was reduced by its small-interfering RNA (siRNA), with raised HMGB1 and apoptosis. Moreover, the phagocytic ability of WT TECs, analyzed by pHrodo *Escherichia coli* bioparticles, was promoted by H_2_O_2_ but inhibited by P^KO^. These results were confirmed by counting phagocytosed H_2_O_2_-induced apoptotic TECs by *in situ* end labeling fragmented DNAs but not affected by additional serum with/without properdin. Taken together, P^KO^ results in impaired phagocytosis at the repair phase post renal IR injury. Properdin locally produced by TECs plays crucial roles in optimizing damaged cells and regulating phagocytic ability of TECs to effectively clear apoptotic cells and reduce inflammation.

## Introduction

Acute kidney injury (AKI) has high morbidity and mortality and also has a potential of developing chronic kidney disease (CKD) (1–3). Renal ischemia–reperfusion (IR) injury, a major cause of AKI, is characterized by circulatory disturbance, complement activation, tubular injury, interstitial inflammation, and eventual fibrosis (4, 5). Tubular epithelial cells (TECs) are most vulnerable to IR injury but also actively participate in repair through dedifferentiation, proliferation, and clearing injured cells by phagocytosis (6–8). However, these functions may be maladaptive in severe and repeated mild injury, resulting in persistent complement activation, inflammation, and fibrosis (9, 10).

Complement activation *via* the alternative pathway (AP) is key to induce renal IR injury in rodent models. The suppression of the AP promotor, complement factor B, using monoclonal antibodies (mAb) or genetic modification significantly preserved renal function and morphology in mice subject to IR injury (11, 12). Conversely, the depletion of the AP inhibitors, decay-accelerating factor (DAF) and CD59, also exacerbates renal IR injury (13).

Properdin, the only known positive regulator of the AP, mainly produced by inflammatory cells (14–17), stabilizes complement 3 (C3) convertase (C3bBb), thereby providing a C3b amplification loop, and then stabilizes C5 convertase (18, 19). Inhibiting properdin by either antimouse mAb or gene deletion in DAF and CD59 double-knockout (DAF^−/−^CD59^−/−^) mice ameliorated early renal IR injury at 24 h (20).

Properdin also functions as a pattern-recognition molecule (PRM) by binding to targets such as damaged cells or bacteria, independent of its main ligand C3b (21–24). Properdin may also bind to the luminal membrane of proximal TECs in the proteinuric kidneys to mediate complement activation (25). However, the precise role of properdin in renal IR injury and repair and its potential function as a PRM in kidneys and TECs are incompletely defined. We hypothesized that properdin may have different roles at different stages of IR injury, either detrimental in the early injury stage by activating AP or beneficial at the late repair stage by facilitating phagocytic clearance of damaged cells.

In this study, properdin knockout (P^KO^) mice and counterpart wild-type (WT) mice were subject to 30 min of bilateral renal ischemia followed by 72 h reperfusion. TCMK-1 cells (mouse kidney cell line) and TECs isolated from P^KO^ and WT mice, respectively, were exposed to hydrogen peroxidase (H_2_O_2_) to mimic the oxidative stress of IR injury. The role of properdin as a PRM and beyond was explored at the repair stage of kidneys post IR and in TECs subjected to IR-related injury, focusing on phagocytosis, apoptosis, and inflammation.

## Materials and Methods

### Animal Model

Properdin-deficient mice were generated by site-specific targeting and maintained at University of Leicester (17). Male C57BL/6 WT and P^KO^ mice aged 8–12 weeks were used in this study. All procedures were performed in accordance with the institutional guidelines reviewed by the Animal Welfare and Ethical Review Body and under the license approved by the UK Home Office (Project License, 70/8169 and Personal License, IA536CDE7). Mice were randomly divided into four groups: (I) WT sham (n = 4), (II) P^KO^ sham (n = 5), (III) WT IR (n = 9), and (IV) P^KO^ IR (n = 7). The animals were anesthetized by inhalation of 2.5% isoflurane in oxygen, and experimental procedures were optimized and refined based upon published protocols (26). The body temperature of animals during surgery was maintained at 36.5°C–37°C. Bilateral renal pedicles were exposed in the lateral position, dissected and clamped with a non-traumatic vascular clip for 30 min. After application of clips, the kidney was observed until patchy blanching developed and then replaced into the abdominal cavity. After removal of clips, gradual appearance of a normal pink color indicated kidney reperfusion.

**Figure 1:**
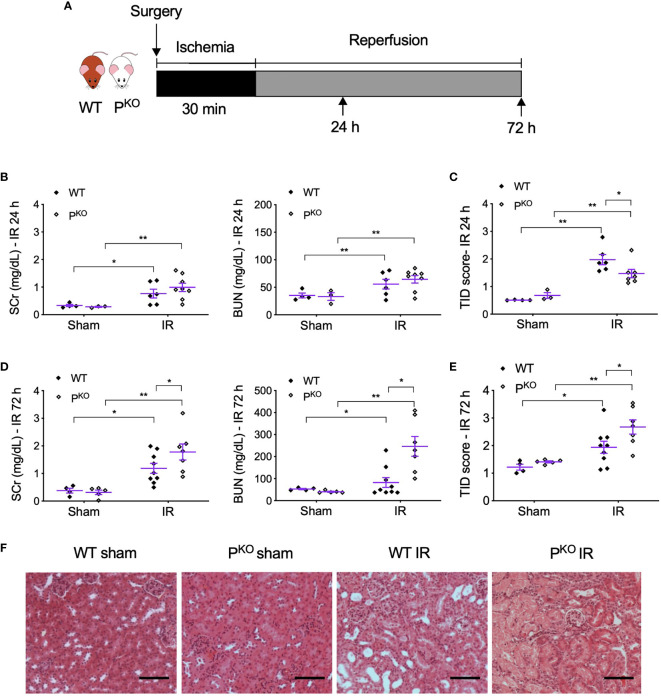
P^KO^ alleviated kidney structural damage at 24 h but aggravated renal functional and structural injury at 72 h post IR. **(A)** Schematic illustration of mouse experimental design. Bilateral kidney ischemia for 30 min and reperfusion for 24 h or 72 h were performed in male WT and P^KO^ C57BL/6 mice. **(B, C)** SCr, BUN, and semiquantitative score of TID in both WT and P^KO^ mice at 24 h were increased by IR, but only TID was improved by P^KO^ (sham: n = 3–4; IR: n = 6–8). **(D, E)** At 72 h, SCr, BUN, and TID score in both WT and P^KO^ mice were also elevated by IR, but all parameters were further increased by P^KO^ (sham: n = 4–5; IR: n = 7–9). Data were shown as means ± SEMs, and analyzed by one-way ANOVA and LSD test. *p < 0.05; **p < 0.01. **(F)** Representative H&E stained images showing the cortex structure of the kidney at 72 h post IR. Scale bar: 100 μm.

After 72 h, the animals were bled by cardiac puncture and then sacrificed. The kidneys were harvested and snap frozen in liquid nitrogen or fixed in 4% (w/v) formaldehyde in 10% normal saline for further analysis. The animal experimental design is shown in **Figure 1A**.

### Renal Function

Serum creatinine (SCr) and blood urine nitrogen (BUN) were determined using the QuantiChrom™ Creatinine or Urea Assay Kit (BioAssay System, Hayward, USA). All procedures were performed according to the manufacturer’s instructions.

### Histology

Paraffin-embedded kidney tissues were sectioned at 4 µm and stained by hematoxylin and eosin (H&E). The scoring of tubulointerstitial damage (TID) and counting of mitotic figures in the cortex was performed blindly to experimental groups by two independent researchers. The criteria of TID scoring included the loss of tubular epithelia, tubular cell vacuolation, tubular dilatation, luminal cast formation, and interstitial expansion (27). A histological score was assigned based on the percentage of affected areas in observed regions: 0, no damage <1%; 1, 1%–25%; 2, 26%–50%; 3, 51%–75%; and 4, >75%. In addition, mitotic figures were counted as condensed chromosomes aligned in metaphase in dividing cells (28). TID and mitotic figures were evaluated in 12 or 20 non-overlapping cortical fields at 200× or 400× magnification, respectively.

### *In Situ* End Labeling of Apoptotic Cells

Apoptotic cells in kidneys were identified by *in situ* end labeling (ISEL) of fragmented DNA using terminal deoxynucleotidyl transferase-mediated uridine triphosphate provided in ApopTag^®^ Peroxidase Kit (Merck Millipore, Darmstadt, Germany) (27). The ISEL+ cells were counted respectively in the tubular areas, tubular lumens, and interstitial areas of the renal cortex of 20 non-overlapping fields at 400× magnification.

### Immunoblotting

Twenty-five micrograms of protein was separated by the electrophoresis of polyacrylamide denaturing gels and transferred onto polyvinylidene fluoride membrane. The primary antibodies, rabbit–antimouse caspase-3 (1:400, 9662, CST, Danvers, USA), rabbit–antimouse high-mobility group box-1 protein (HMGB1, 1:1,000, 3935, CST), mouse–antimouse proliferating cell nuclear antigen (PCNA, 1:1,000, M0879, DAKO, Glostrup, Denmark), rabbit–antimouse erythropoietin receptor (EPOR, 1:1,000, PAB18350, Abnova, Taiwan), rabbit–antimouse properdin (1:1,000, AB186834, Abcam, Cambridge, USA) and mouse–antimouse β-actin (1:5,000, A5441, Sigma, Dorset, UK), were applied to the membranes overnight at 4°C, followed by the incubation of horseradish peroxidase-labeled secondary antibody (goat–antirabbit/mouse, K4063, DAKO) and developed by enhanced chemiluminescence (Thermo Fisher Scientific, Rockford, USA). The ratio of target protein to β-actin in volume density, as an endogenous loading control, was calculated for each detection, and then, the fold change of detected protein in the experimental group against the WT sham control was obtained as final results (27).

### Immunohistochemistry

Antigen retrieval was performed using 20–40 µg/ml proteinase K (Sigma) digestion at 37°C for 15 min. The sections were then blocked with 10% goat serum and 0.5% bovine serum albumin (BSA) in 3% milk for 1 h at room temperature, followed by primary antibody labeling, mouse–antimouse F4/80 (1:100, ab100790, Abcam) or rabbit–antimouse erythropoietin receptor (EPOR, 1:800, PAB18350, Abnova) at 4°C overnight. The next day, DAKO secondary (K4063) was applied to the sections for 30 min. The antibody binding was then revealed by 3,3′-diaminobenzidine (DAB, Vector, Burlingame, USA) and hematoxylin counterstaining.

For double labeling of properdin and F4/80 in the kidneys of WT mice, proteinase K at 40 µg/ml was used for antigen retrieval at 37°C for 30 min. After the staining of F4/80 was obtained, the sections were then incubated with primary antibody rabbit–antimouse properdin (1:200, ABF185, Merck Millipore) at 4°C overnight. Afterwards, biotinylated secondary goat–antirabbit immunoglobulin G (IgG) was applied to the slides for 30 min at 37°C (1:300, BA-1000, Vector), followed by alkaline phosphatase streptavidin (1:200, SA-5100, Vector) for 30 min at 37°C, and then developed by Fast Red (Sigma) for 8 min.

### Real-Time Quantitative PCR

Total RNA was extracted using Trizol reagent (Thermo Fisher Scientific). Three micrograms of total RNA was used for reverse transcription (Thermo Fisher Scientific). One microliter of complementary DNA (cDNA) product was amplified with SYBR Green reaction system (Bioline, London, UK) containing 250 nM forward and reverse primers (Sigma, **Table 1**) at 95°C for 10 min followed by 40 cycles of 95°C for 20 s, 60°C for 30 s, and 72°C for 20 s. The level of glyceraldehyde 3-phosphate dehydrogenase (GAPDH) messenger RNA (mRNA) was detected as an endogenous control.

**Table 1:** The sequence of primers for real-time quantitative PCR.

Genes	Primers (5′–3′)
Properdin	Forward: TTCACCCAGTATGAGGAGT Reverse: GCTGACCATTGTGGAGACCT
GAPDH	Forward: CCTGGAGAAACCTGCCAAGTATG Reverse: AGAGTGGGAGTTGCTGTTGAAGTC

### Double Labeling of Apoptotic TCMK-1 Cells With Properdin

TCMK-1 cells (American Type Culture Collection, Manassas, USA), mouse kidney epithelial cell line, were grown in Dulbecco’s modified Eagle’s medium (DMEM)/F-12 medium (Gibco, Carlsbad, USA) with 10% (v/v) fetal calf serum (Gibco), 2 mM L-glutamine (Gibco), 100 U/ml penicillin G, and 100 mg/ml streptomycin (Sigma, Dorset, UK) at 37°C in a 5% CO_2_ humidified atmosphere.

TCMK-1 cells were cultured in serum-free DMEM/F-12 medium (Gibco, Carlsbad, USA) and stimulated with 200 μM H_2_O_2_ for 24 h. The cells were then fixed and subjected to ISEL and developed with 3,3′-diaminobenzidine (DAB) chromogen (Vector). Antiproperdin antibody (ab186834, Abcam) diluted 1:100 was applied followed by biotinylated goat–antirabbit IgG (1:300, BA-1000, Vector) and alkaline phosphatase streptavidin (1:200, SA-5100, Vector). Binding was detected by Fast Red (Sigma) and hematoxylin counterstaining. For the negative control, the primary antibody was substituted with normal rabbit IgG of same species (Merck Millipore) at same protein concentration.

### TCMK-1 Cells Treated by Properdin siRNA

Cells were seeded onto six-well plates at 2 × 10^5^ density. When 50% confluence was reached, the cells were transfected with small interfering RNA (siRNA) targeting properdin (siP, Thermo Fisher Scientific) or negative control siRNA (siNC, does not target any known mammalian genes) at 16 nM, assisted with Lipofectamine™ RNAiMAX (Invitrogen, Carlsbad, USA). After 6 h transfection, the cells were treated with 200 μM H_2_O_2_ and then lysed at 18 h to extract whole protein for detecting properdin and HMGB1 by Western blotting. In addition, the number of apoptotic cells was evaluated by Annexin V/PI staining (Roche, Mannheim, Germany) according to the manufacturer’s instruction and then detected by a flow cytometer (BD, Bergen, USA). Briefly, TCMK-1 cells were tripsinized, resuspended in binding buffer, and incubated with fluorescein isothiocyanate (FITC)-conjugated Annexin-V and propidium iodide (PI) for 15 min. Cells (10,000) were analyzed by BD FACS Calibur flow cytometry (BD Biosciences, Franklin Lakes, USA). Living cells (Annexin-V−/PI−, lower left quadrant), early apoptosis (Annexin-V+/PI−, lower right quadrant), late apoptosis (Annexin-V+/PI+, upper right quadrant), or necrosis (Annexin-V−/PI+, upper left quadrant) were shown as quadrant dot plots and the percentage of the gated cells (29). In each experiment, two replicates per group were used, while the individual experiment was repeated at least three times.

### Primary Isolated TECs From WT and P^KO^ Mice

The TECs were primary isolated from the kidneys of both WT and P^KO^ C57BL/6 male mice aged 8–12 weeks using a well-established method that led to the functional characterization of TECs in the group (30). Briefly, small cortical pieces from the kidney were pulverized and passed through a serial set of sieves with the size from 250, 125, 75, and 45 μm. The primary isolates were maintained in DMEM/F-12 medium containing 10% heat-inactivated fetal bovine serum (FBS, Sigma) and other basic additives as the same for TCMK-1 cells, but with additional recombinant human epithelial growth factor (0.1 µg/ml), insulin (5 µg/ml), transferrin (5 µg/ml), sodium selenite (5 ng/ml), triiodothyronine (4 pg/ml), and hydrocortisone (36 ng/ml). At confluence, TECs (passage 0) were split using trypsin/ethylenediaminetetraacetic acid (EDTA) (Invitrogen), and cells at passages 2–3 were used for experiments. The characterization of isolated TECs was previously validated by the positive staining of cytokeratin and γ-glutamyl transpeptidase and the negative staining of factor VIII and α-smooth muscle actin that are the markers of endothelial cells and mesangial cells, respectively (30). In addition, the morphological characteristics of newly isolated and cultured TECs that formed domes are illustrated in **Supplementary Figure 1** (31).

### Assessing Phagocytosis of WT and P^KO^ TECs

*Escherichia coli (E. coli)* bioparticles (FITC-labeled pHrodo *E. coli* Bioparticles^®^ Conjugate P35366, Thermo Fisher Scientific) were used to assess the phagocytic ability of WT and P^KO^ TECs by flow cytometry. Primary isolates of WT and P^KO^ TECs at passage 2 or 3 were seeded into a 24-well plate at 1.8 × 10^5^ and cultured in the complete medium containing 10% heat-inactivated FBS. Following attachment, the cells were then exposed to 200 µM H_2_O_2_ for 24 h and followed by incubation with 500 µl of *E. coli* bioparticles (0.5 mg/ml, suspended in DMEM/F12 medium) for 2 h. Non-phagocytosed *E. coli* bioparticles were removed, and the cells were trypsinized and resuspended. On a fluorescence-activated cell sorting (FACS) system (BD), a total of 10,000 gated cells were analyzed for the fluorescent intensity of FITC. Due to the different level of auto fluorescence in WT TECs and P^KO^ TECs, a different threshold was set up for each phenotype. To make two phenotypes comparable, the fold change of positive cells or FITC fluorescent intensity of total cells was calculated against that of the control—*E. coli* group for each phenotype.

The primary cultured WT TECs and P^KO^ TECs at passage 2 or 3 were also seeded onto glass coverslips precoated with poly-D-lysine (0.1 mg/ml; Sigma) and cultured in the above-described culture medium, either without serum or with 2% serum from WT or P^KO^ mice without heat inactivation for 24 h. The cells were stimulated with 200 μM H_2_O_2_ for another 24 h. The TECs were fixed by 1% paraformaldehyde for 10 min and then by an ice-cold mixture of ethanol and acetic acid (2:1) at −20°C for 5 min, followed by ISEL staining. The number of ISEL+ cells, phagocytosed ISEL+ cells (normal TEC overlapping with ISEL+ cells), and total cells were counted in 20 random fields at 400× magnification.

### Statistical Analysis

Data are expressed as mean ± standard error of the mean (SEM). One-way ANOVA was used to check the homogeneity of variance, followed by *post-hoc* least significant difference (LSD) test for multiple comparisons or unpaired Student’s t-tests for comparison between two groups, using SPSS Statistics Standard V26.0 software (IBM, New York, USA). *p* < 0.05 was considered statistically significant.

## Results

### P^KO^ Preserved Kidney Structure at Early Stagy but Aggravated Kidney Damage at Late Phase Post IR

To differentiate whether properdin has different roles at the different stages of IR injury, we established bilateral renal IR model in mice with 30 min ischemia followed by reperfusion for 24 h as an early predominate injury stage and 72 h for a late kidney repair stage. The experimental design was shown in **Figure 1A**. Compared to sham controls, SCr, BUN, and TID score in H&E-stained sections were elevated by IR in both WT and P^KO^ mice at 24 h (**Figures 1B, C**), with significantly lower TID score in IR P^KO^ kidneys than WT controls. At 72 h, SCr, BUN, and TID score were also elevated by IR in both WT and P^KO^ mice, while, notably, all these parameters were significantly higher in P^KO^ mice than WT controls (**Figures 1D–F**).

### P^KO^ Increased Apoptotic Cells in Tubular Luminal Areas Post 72-h IR

**Figure 2:**
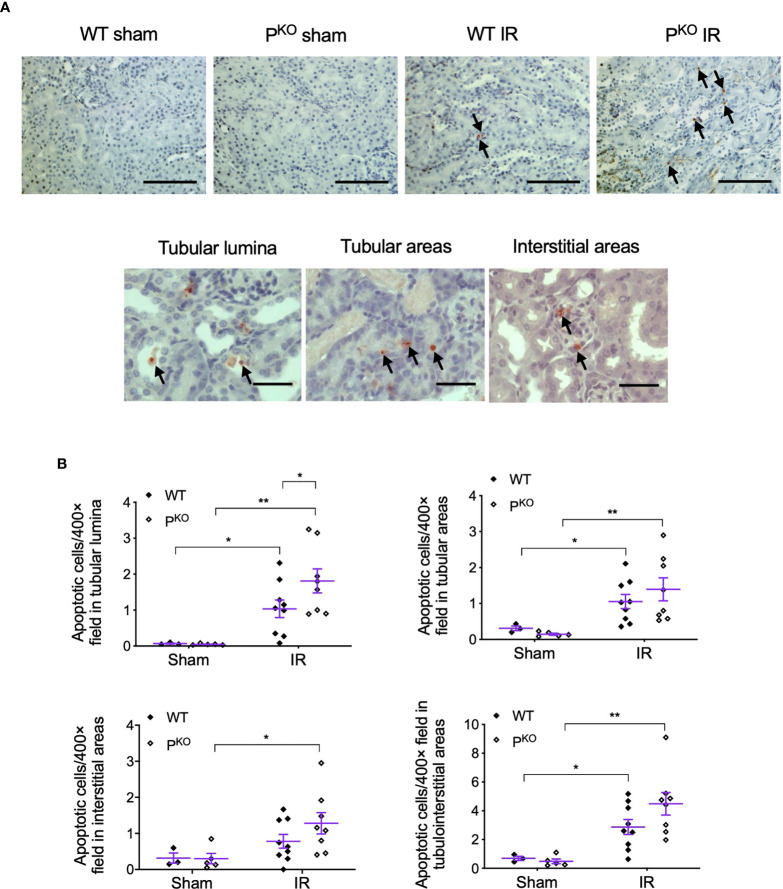
P^KO^ increased the number of apoptotic cells in tubular lumina post IR at 72 h. **(A)** Representative photomicrographs showed apoptotic cells in the indicated groups and tubular lumina, tubular and interstitial areas, revealed by ISEL with AEC (3-amino-9-ethylcarbazole). All images were taken from the renal cortex. Scale bar: 100 μm (upper row) and 50 μm (lower row). **(B)** The number of ISEL+ cells in tubular lumina was increased by IR and furthered by P^KO^, whereas in the area of tubule, interstitium and tubulointerstitium P^KO^ caused a slightly higher level of apoptosis than the WT control but did not reach statistical significance. Data were shown as means ± SEMs and analyzed by one-way ANOVA and LSD test. *p < 0.05; **p < 0.01.

As properdin has potential PRM function of labeling apoptotic cells for their recognition by phagocytes, absence of properdin could result in delayed phagocytosis of apoptotic cells and the accumulation of these cells in injured kidneys. Thus, the level of renal apoptosis was assessed by *in situ* labeling of apoptotic cells in IR kidneys of both WT and P^KO^ mice. Apoptotic cells with typical morphologic features, detected by ISEL-fragmented DNAs, mainly distributed in tubulointerstitial areas (**Figure 2A**), were dramatically increased by IR in both WT and P^KO^ kidneys, but were scant in sham kidneys (**Figure 2B**). In the tubular lumen, apoptotic cells were significantly greater in IR P^KO^ kidneys compared with WT kidneys. Similarly, apoptotic cells in tubules and interstitial areas were increased by IR in WT and P^KO^ kidneys, without significant differences between phenotypes.

### P^KO^ Increased Inflammation and Its Mediators in Kidneys Post 72-h IR

**Figure 3:**
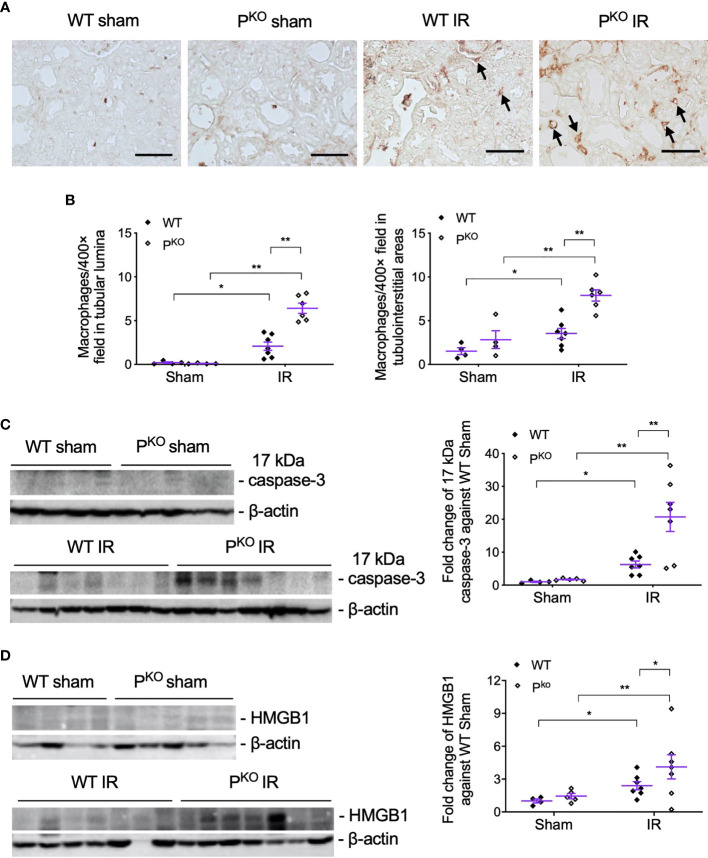
P^KO^ increased the infiltration of inflammatory cells and the expression of injury markers post IR at 72 h. **(A, B)** Representative images of F4/80 immunostaining in the indicated groups, and the semiquantitative analysis, showed more F4/80+ cells in the interstitium and tubulointerstitium of P^KO^ kidneys than WT controls (sham: n = 4; IR: n = 6–7). The images were taken from the cortical area of the kidney. Scale bar: 50 μm. **(C, D)** Seventeen kilodaltons active caspase-3 and HMGB1 protein in the kidney determined by Western blot was increased by IR and furthered by P^KO^ (sham: n = 4-5; IR: n = 7–9). Data were shown as means ± SEMs and analyzed by one-way ANOVA and LSD test. *p < 0.05; **p < 0.01.

P^KO^-associated more severe IR kidney damage at 72 h was further examined closely by assessing infiltrated inflammatory cells in kidneys by the immunostaining of F4/80, a marker of macrophages, and examining injury markers including 17 kDa active caspase-3 and HMGB1 in kidney homogenates using Western blot. F4/80+ macrophages in tubular lumina and tubulointerstitial areas were remarkably increased by IR compared to sham groups, and further elevated by P^KO^ (**Figures 3A, B**). The expression of 17 kDa active caspase-3, an executer of apoptosis (32) and inflammation (33, 34), was increased by IR compared to sham controls of WT and P^KO^ mice, and further raised in IR kidneys by P^KO^ compared to WT (**Figure 3C**). The same change pattern was also revealed in HMGB1 protein (**Figure 3D**), a recruiter of inflammatory cells and activator of complement (35–38).

### IR Raised Mitosis and PCNA With P^KO^ Increased EPOR Post 72-h IR

**Figure 4:**
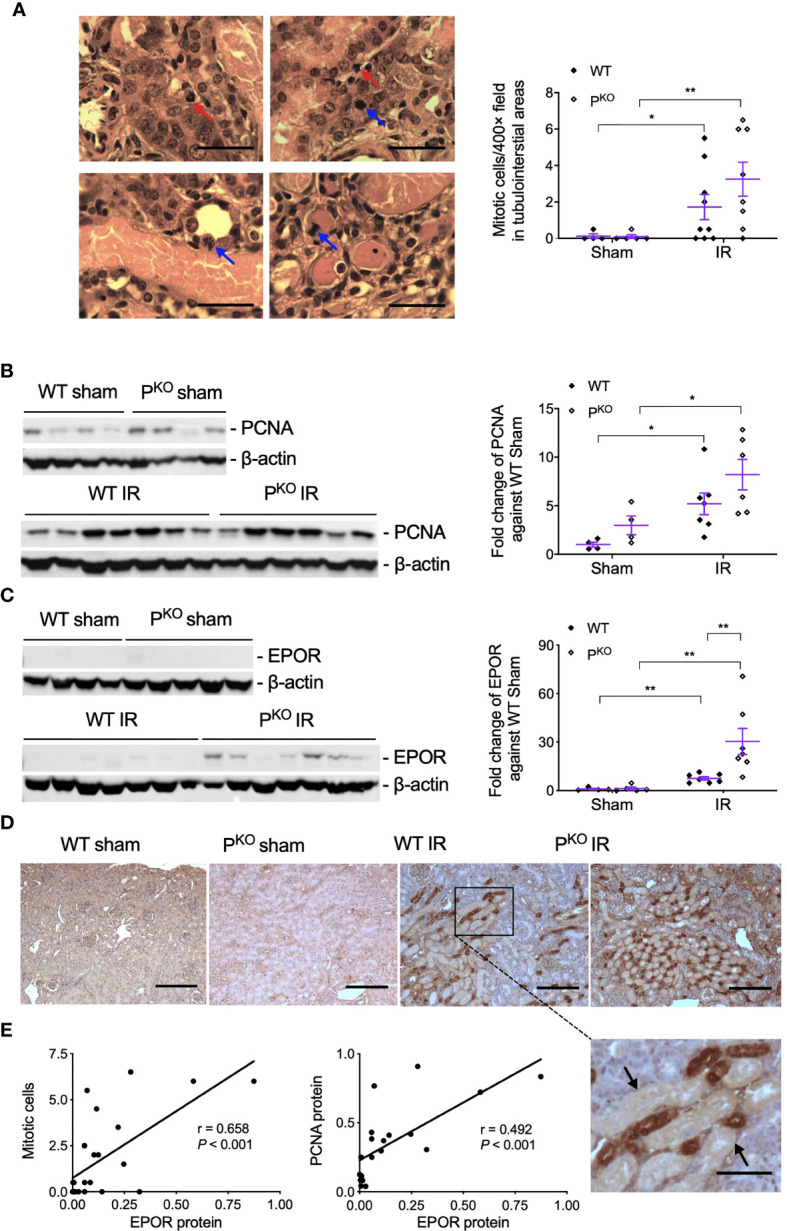
Kidney repair parameters in P^KO^ kidneys post IR at 72 h. **(A)** Mitotic cells with typical morphological features in tubular and interstitial areas were pointed by blue and red arrows (upper panels, WT IR kidneys; lower panels, P^KO^ IR kidneys). The number of mitotic cells was increased by IR in both WT and P^KO^ mice assessed by semiquantitative analysis. Scale bar: 20 μm. **(B)** The expression of PCNA protein in the kidney determined by Western blot was increased by IR in both WT and P^KO^ mice. **(C)** The expression of EPOR protein was also increased in the IR kidneys of both genotypes with a further elevation by P^KO^ compared to WT controls. Data were shown as means ± SEMs and analyzed by one-way ANOVA and LSD test. *p < 0.05; **p < 0.01. **(D)** Representative images of EPOR immunostaining in the cortex of kidneys, and the area from a dashed box was enlarged and necrotic tubules were pointed by arrows. Scale bar: 100 and 50 μm (the enlarged image). **(E)** The linear correlation showed EPOR protein positively correlated to mitosis and PCNA, respectively.

As repair was initiated at 72 h post IR, the compensatory response under the absence of properdin was assessed by mitotic cells and the proliferative marker PCNA. Mitotic cells in H&E-stained sections, reflected kidney repair, were increased by IR in WT and P^KO^ kidneys compared to the phenotype sham (**Figure 4A**), while a similar change was seen in PCNA protein detected by Western blot, without significant differences between P^KO^ and WT (**Figure 4B**).

To further understand the role of P^KO^ in the repair phase post IR, the expression and localization of EPOR, a part of innate repair receptor (39) EPOR/βcR and essential for the phagocytic function of macrophages (40), were evaluated by Western blot and immunostaining. EPOR was increased by IR in WT and P^KO^ kidneys compared to respective sham controls, further elevated by P^KO^ compared to WT (**Figure 4C**). The weak staining of EPOR was evenly distributed in the cytoplasm of TECs in the cortical areas of sham kidneys, greatly enhanced by IR, mainly localized around severely damaged tubules, in WT and P^KO^ mice (**Figure 4D**). The level of EPOR was positively correlated with mitotic figures and PCNA (**Figure 4E**).

### Elevated Properdin in IR Kidneys Was Localized in TECs and Macrophages

**Figure 5:**
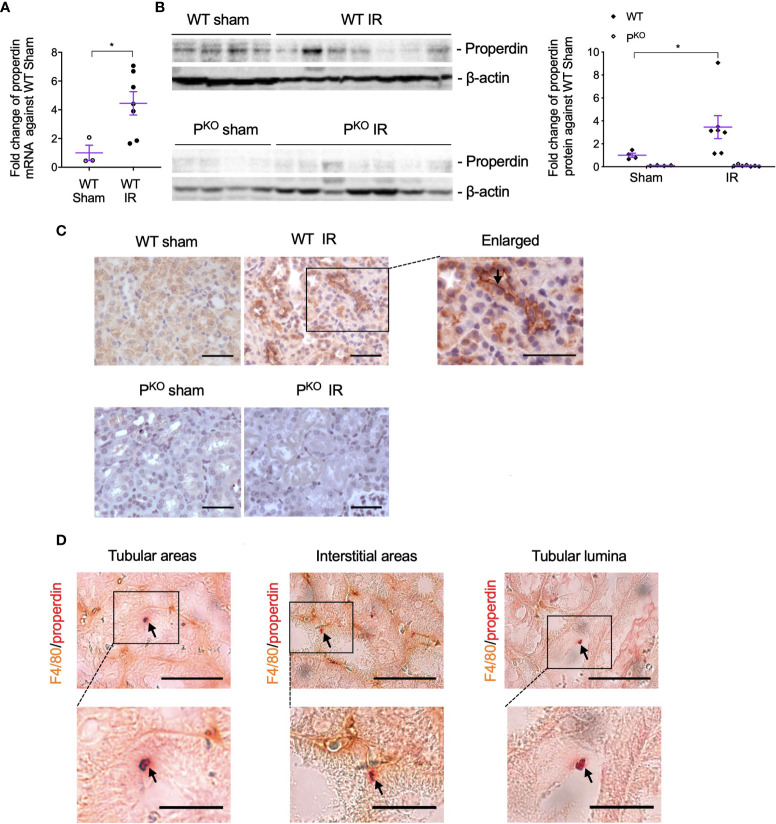
Properdin expression and localization in kidneys post IR at 72 h, as well as its co localization with F4/80+ macrophages. **(A)** The expression of properdin mRNA in kidneys analyzed by qPCR was raised by IR in contrast to the sham control in WT mice. **(B)** The expression of properdin protein in kidney homogenates detected by Western blot was increased by IR in WT mice but was not seen in P^KO^ mice (sham: n = 3–4; IR: n = 7). Data were shown as means ± SEMs. Significance was assessed by unpaired *t*-test for qPCR result and one-way ANOVA and LSD test for Western blots. *p < 0.05. **(C)** Representative images of properdin immunostaining in the cortex of WT sham and IR kidneys, with the boxed area enlarged and properdin staining on the apical surface of tubular cells was pointed by an arrow. All scale bar represent 50 μm. **(D)** Properdin and F4/80 double-stained cells were visualized in tubular areas, interstitial areas, and tubular lumina (pointed by arrows), with the boxed area enlarged. Most of double staining positive cells had typical apoptotic features including reduced cell volume, and the cellular membrane becoming ruffling and blebbing were also shown. Scale bar: 50 μm (upper row) and 20 μm (lower row).

To observe the involvement of properdin in IR injury and whether properdin tags apoptotic inflammatory cells, the expression of properdin mRNA and protein in IR kidneys was first examined and then followed by its double immunostaining with F4/80 proteins. Exons 3–7 of properdin DNA was absent in P^KO^ mice (17), so properdin mRNA was detected by quantitative PCR (qPCR) using primers recognizing properdin inside the exons 3–7. The level of properdin mRNA was raised by IR compared to sham controls in WT kidneys (**Figure 5A**), in which properdin protein was also elevated, but absent in P^KO^ kidneys (**Figure 5B**).

The immunostaining of properdin was mainly localized on the apical surface of TECs and raised by IR in WT kidneys (**Figure 5C**). Properdin and F4/80 double-stained cells with typical apoptotic morphological features were observed in tubular areas, interstitial areas, and tubular lumina in IR WT kidneys (**Figure 5D**).

### Properdin Tagged Apoptotic Cells Phagocytosed by Healthy Cells

**Figure 6:**
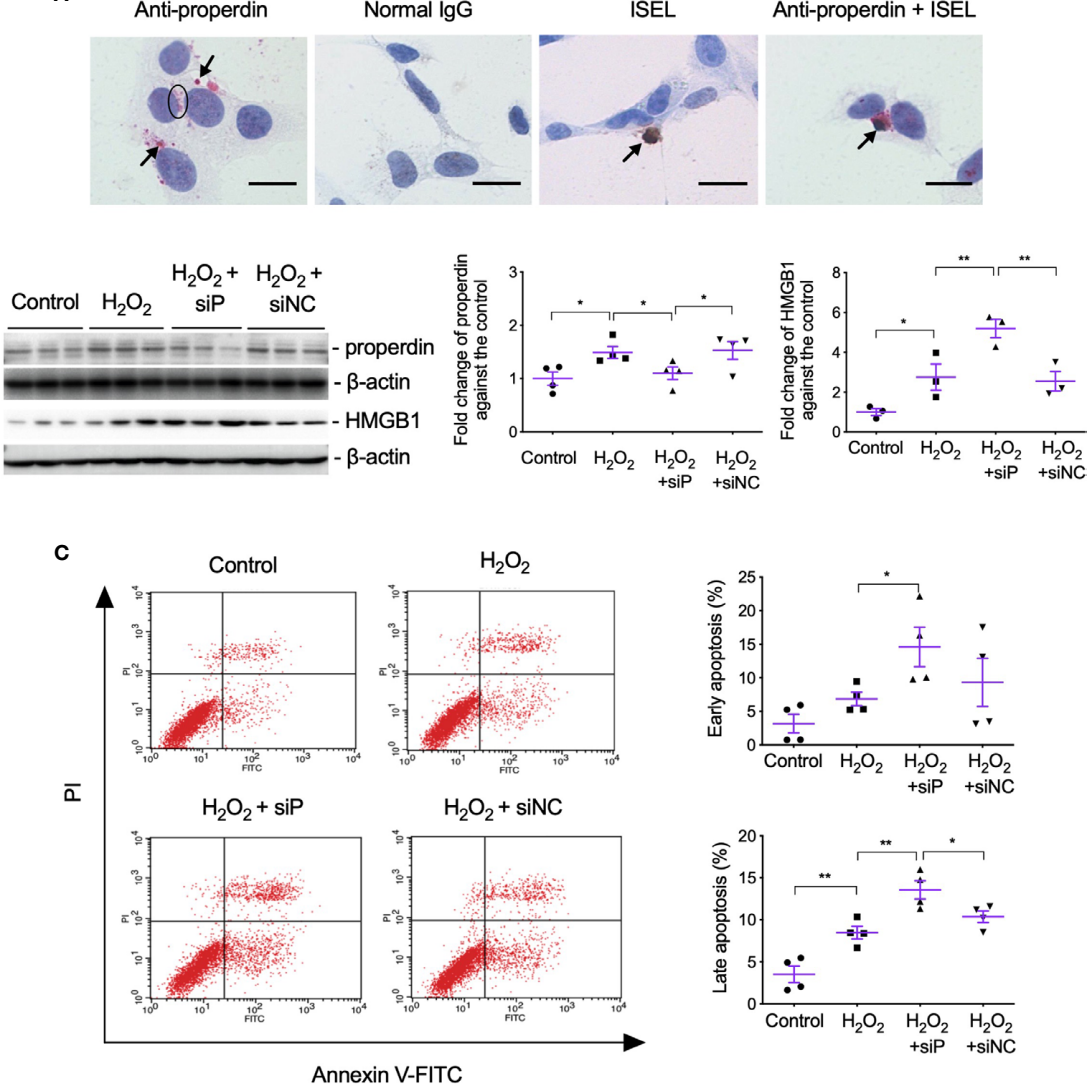
Properdin tagged apoptotic TCMK-1 cells and inhibiting properdin increased apoptotic cells and HMGB1 expression in H_2_O_2_-treated TCMK-1 cells. **(A)** Properdin protein was visualized in TCMK-1 cells after H_2_O_2_ treatment for 24 h (indicated by a circle and two arrows nearby), in which the staining of properdin was negatively controlled by normal rabbit IgG. ISEL+ apoptotic TCMK-1 cells and its colocalization with properdin protein were also seen (pointed by another two arrows, n = 3). Scale bar: 5 μm. **(B)** The expression of properdin and HMGB1 detected by Western blot was increased by H_2_O_2_ treatment for 24 h, whereas siP reduced properdin expression but increased HMGB1 (n = 3). **(C)** The early and late apoptotic TCMK-1 cells detected by flow cytometry analysis were both increased by H_2_O_2_ treatment for 24 h and furthered by siP (n = 3). FITC fluorescein was tagged to Annexin V, thus can reveal the binding of Annexin V to the cellular surface of apoptotic cells that exposed phosphatidylserine (PS). Propidium iodide (PI) passed leaky necrotic cells to stain the DNA. Data were shown as means ± SEMs and analyzed by one-way ANOVA and LSD test. siP, siRNA targeting properdin; siNC, negative control siRNA. *p < 0.05; **p < 0.01.

To further examine the effect of properdin as a PRM on apoptotic renal parenchymal cells, the double staining of properdin and ISEL was performed in H_2_O_2_-treated TCMK-1 cells *in vitro*. Patchy properdin staining was observed mainly in the cytoplasm of cells, without any staining in negative control cells incubated with normal rabbit IgG (**Figure 6A**). Some properdin+ cells having typical apoptotic features, shrunken nuclei with halos and condensed cytoplasm, or properdin and ISEL double+ cells, and ISEL+ cells were adjacent to or phagocytosed by healthy cells.

The effect of properdin on TECs was further verified by siP in H_2_O_2_-treated TCMK-1 cells, in which siP downregulated properdin expression but further increased HMGB1 (**Figure 6B**) and the number of early and late apoptotic cells in contrast to the negative control (siNC, **Figure 6C**).

### Phagocytic Ability of TECs Reduced by P^KO^

**Figure 7:**
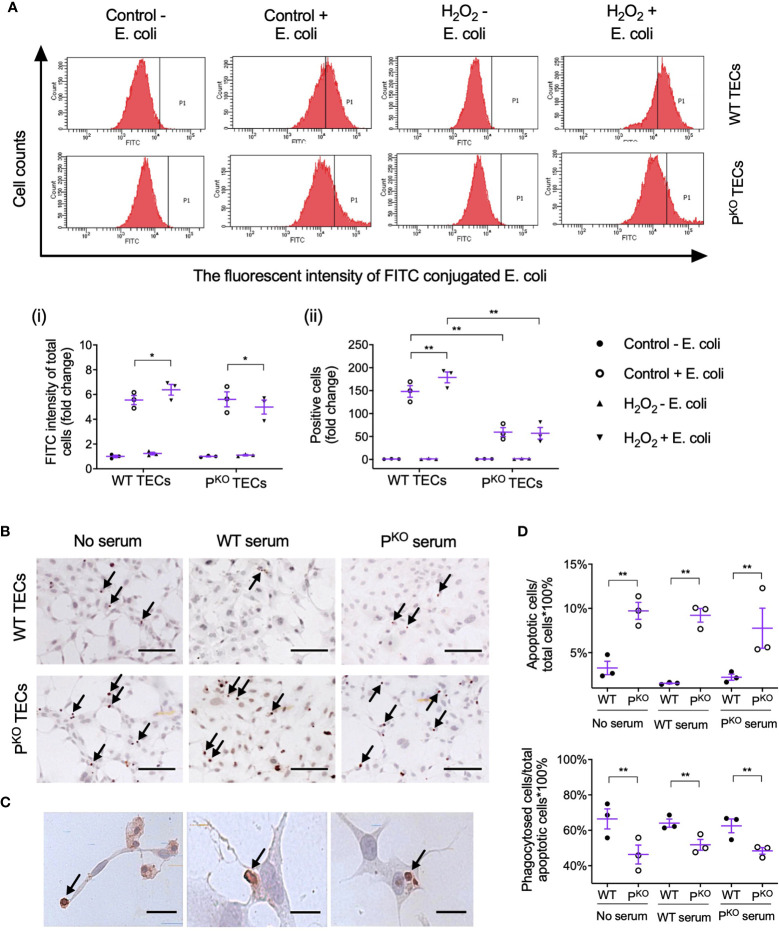
Properdin contributes to the phagocytic efficacy of primary isolated TECs. **(A)** Flow cytometry analysis of phagocytosed FITC-labeled *E. coli* by primary isolated WT and P^KO^ TECs with or without H_2_O_2_ treatment for 24 h (n = 3). The fluorescent intensity of FITC conjugated *E. coli* reflected the phagocytic function of TECs. (i) The fold change of the average intensity of FITC fluorescence among total cells, calculated against the control (−*E. coli*) group, was increased in WT TECs but decreased in P^KO^ TECs by H_2_O_2_. (ii) The fold change of *E. coli* positive cell counts (P1 area) in each group against the control (−*E. coli*) group was increased in WT TECs by H_2_O_2_ but stayed low in P^KO^ TECs of either the control or H_2_O_2_ groups compared to corresponding WT groups. **(B)** Representative images of apoptotic WT and P^KO^ TECs detected by ISEL (ISEL+ cells indicated by arrows, n = 3). Scale bar: 50 μm. **(C)** Healthy WT TECs approaching, partially engulfing, and phagocytosing adjacent ISEL+ cells were demonstrated and indicated by arrows. Scale bar: 5 μm. **(D)** Semiquantitative analysis showed that the percentage of ISEL+ cells against the total number of cells was increased by P^KO^ regardless of serum conditions, while the percentage of phagocytosed ISEL+ cells against the total number of ISEL+ cells was decreased by P^KO^ after H_2_O_2_ treatment for 24 h (n = 3). Data were shown as means ± SEMs and analyzed by one-way ANOVA and LSD test. *p < 0.05; **p < 0.01.

Lastly, whether properdin affects the phagocytic ability of TECs was assessed by flow cytometry using pHrodo *E. coli* bioparticles. The threshold of fluorescent intensity for WT TECs was set lower than that for P^KO^ TECs, as P^KO^ had higher auto fluorescent intensity (Control - *E. coli*, **Figure 7A**), which was not changed by H_2_O_2_ treatment (H_2_O_2_ - *E. coli*). The fold change of FITC fluorescent intensity in all gated cells was increased by H_2_O_2_ only in WT TECs compared to the controls but decreased by P^KO^ (**Figure 7A**, i), which were also seen in the fold change of positive cells (**Figure 7A**, ii). Moreover, compared to WT TECs, P^KO^ decreased the fold change of FITC fluorescent intensity treated by H_2_O_2_ and also decreased the fold change of positive cells without H_2_O_2_.

Apoptotic TECs were approached, engulfed, and phagocytosed by adjacent healthy-appearing TECs (**Figures 7B, C**). The percentage of apoptotic cells against the number of total cells were increased by P^KO^ compared with WT TECs, regardless of serum conditions (**Figure 7D**). However, the percentage of phagocytosed apoptotic cells against the number of apoptotic cells in the P^KO^ TECs was lower than that in WT TECs under all serum conditions.

## Discussion

**Figure 8:**
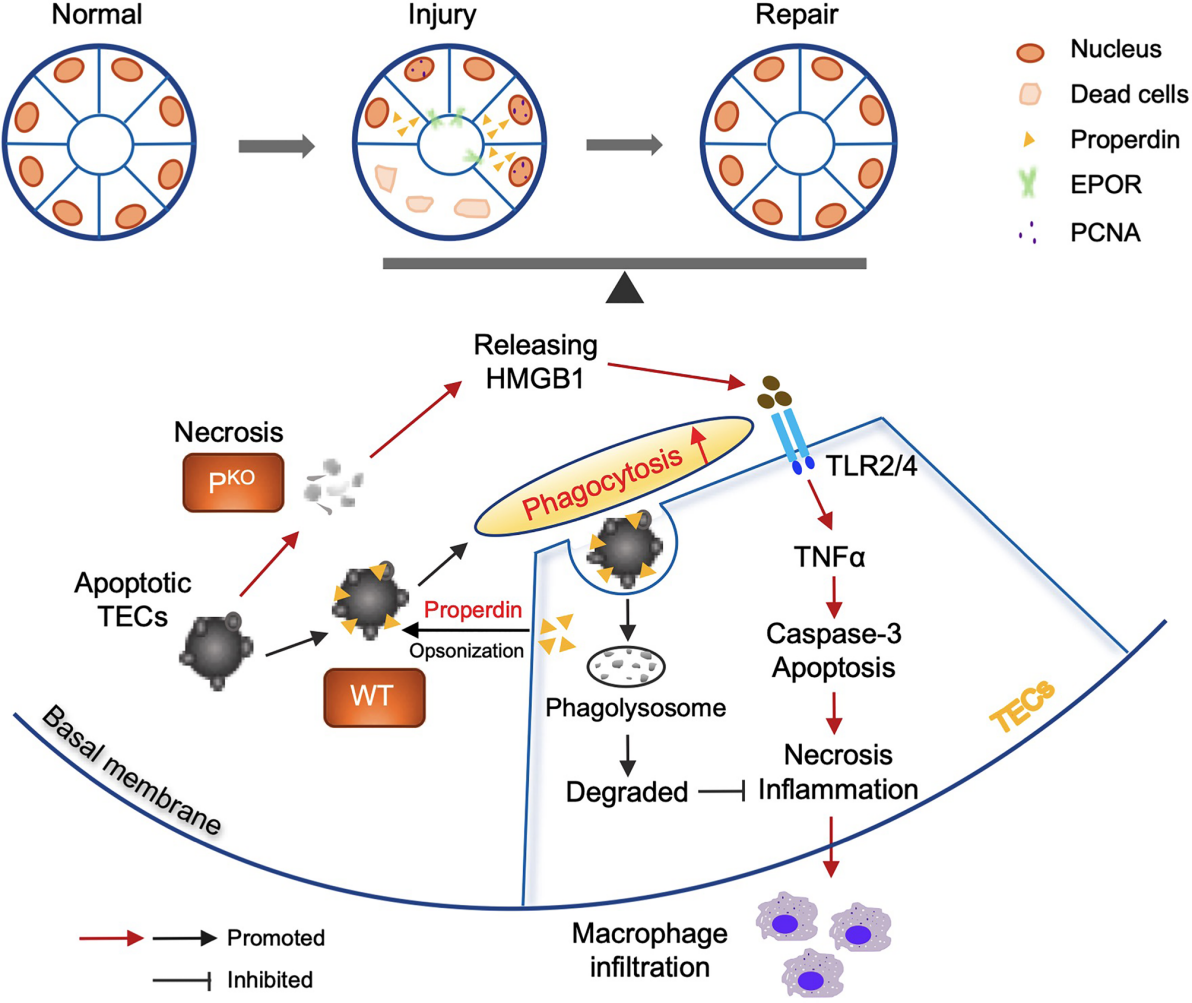
Properdin mediated and enhanced phagocytosis of TECs. Schematic illustration of the role of properdin in the phagocytosis of living TECs from WT and P^KO^ mice. Properdin produced by living TECs tags adjacent apoptotic TECs to facilitate their uptake by neighbor living TECs, promoting damage reduction and inflammatory clearance. Lack of properdin impaired TECs phagocytosing damaged cells and resulted in more severe inflammation and tissue damage and compromised repair activities. Thus, properdin associated phagocytic function of TECs facilitates the balance between injury and repair of the kidney.

Properdin has diverse and disease-dependent effects that may be modified by the genetic background of animal models (20, 41). In 24-h post renal IR mice, depleting negative regulators of the AP increased properdin and AP activation and TEC injury, whereas inhibiting properdin ameliorated renal IR injury (20, 42). Conversely, P^KO^ aggravates C3 glomerulopathy in mice (43, 44). In addition, emerging evidence indicates that properdin tagging apoptotic/necrotic T cells leads to their uptake by phagocytes *via* C3b opsonization or independent of C3b (22). The present study using sole P^KO^ mice demonstrates that properdin has beneficial effects at 72-h kidney repair stage post IR injury, which was attributed to properdin regulating the phagocytic activity of TECs apart from as a PRM optimizing damaged cells (**Figure 8**).

More severe damage in the P^KO^ IR kidneys at 72 h was associated with increased apoptotic cells in tubular luminal areas, and elevated active caspase-3 and HMGB1. Caspase-3 executes apoptosis and activates inflammatory mediators, while HMGB1 activates the Toll-like receptor 4 (TLR4)/nuclear factor kappa B (NF-κB) signaling pathway (45) and cytokine/chemokine production (35–38). P^KO^ resulted in persistent apoptosis and inflammation in the IR kidneys due to absence of properdin-assisted phagocytic clearance of apoptotic cells (22, 46, 47). The accumulation of apoptotic cells in the tubular lumen of P^KO^ IR kidneys may represent an adaptive mechanism of direct elimination, while recruited macrophages may phagocytose apoptotic cells and also penetrate through tubular epithelia into the lumen. Properdin-labeled apoptotic macrophages were shown in the different compartment of IR kidneys of WT mice in this study, thus rendering them susceptible to phagocytes *in vivo*.

EPOR is expressed on TECs, where it forms heterodimers with βcR (EPOR/βcR), an innate repair receptor (39), and ameliorates renal damage and promotes tissue self-repair following IR (27, 48). Distributed mainly around damaged tubules (49), EPOR maintains tissue homeostasis by inhibiting the proinflammatory function of macrophages and enhancing their phagocytic function (40, 50). Increased EPOR, therefore, may also represent the compensatory response of repair in P^KO^ IR kidneys at 72 h.

TECs serve as major local semiprofessional phagocytes (8) and express various complement components, with C3 and factors H, B, I, and D being the most predominant (42, 51). Here, properdin was expressed in renal TECs being protective in terms of limiting HMGB1 and apoptosis under oxidative stress. Properdin, released from TECs, binds to the glycosaminoglycan chains of cell surface proteoglycans in apoptotic TECs to facilitate healthy TECs uptake these cells (22). Effectively clearing apoptotic cells by phagocytosis is crucial to limit renal injury and at the same time promote repair/remodeling post IR (8, 52, 53), in which the process of opsonizing damaged cells was absent in P^KO^ kidney resulting in higher level of apoptosis and severer damage.

Additionally, the effect of properdin on the phagocytic ability of TECs was detected by the uptake of fluorescence labeled *E. coli* particles, and oxidative-stress-induced apoptotic cells. Under unstressed and oxidative-stressed condition, P^KO^ significantly reduced the number of phagocytic cells, which indicates that properdin might affect the transformation of TECs to phagocytes. Moreover, P^KO^ significantly decreased the fluorescence intensity of gated P^KO^ TECs under oxidative stress, which was increased in WT TECs, indicating that properdin is essential for upregulating the phagocytic ability of TECs under stress.

P^KO^ may affect the expression of PRM including properdin per se and pattern-recognition receptors (PRRs) on TECs. Apart from certain “eat me” signals expressed on damaged cells, the PRM further facilitates their recognition by phagocytes. Properdin might also be a constitutive “eat me” marker as it was expressed on the plasma membrane of TECs. For instance, the molecule of kidney injury molecule 1 (KIM-1) is one of the PRRs, expressed on TECs (as semiphagocytes) subjected to injury. KIM-1 as a receptor binds to its ligand, phosphatidylserine, as an “eat me” marker exposed on damaged cells (8). The evidence of reduced phagocytosis in P^KO^ TECs indicates that properdin might not only tag apoptotic cells mediating phagocytosis but also directly affect the phagocytic ability of TECs. In addition, the phagocytic feature of WT and P^KO^ TECs was not affected by additional serum with or with properdin, indicating that only locally produced properdin was essential for phagocytosis. Taken together, this study demonstrates for the first time that properdin has a key direct role in the phagocytic ability of TECs.

There are certain limitations in this study. First, the interaction and mechanism of properdin, KIM-1 and EPOR on the phagocytosis of TECs, and macrophages in renal injury and repair are worthy of further investigation. Second, a time-course model of renal IR in P^KO^ mice will be useful to disclose the dynamic change and the precise role of properdin in injury, recovery, or chronic progression.

In conclusion, this study showed that lack of properdin had harmful effects on kidneys at the repair stage post IR injury, which was attributed to properdin not only opsonizing damaged cells but also affecting the phagocytic ability of TECs to effectively clear apoptotic cells and subsequent inflammation. Therefore, a novel mechanism in IR injury and repair was found, in which properdin is crucial for phagocytosis in the repair stage after IR-induced injury.

## Data Availability Statement

The original contributions presented in the study are included in the article/**Supplementary Material**. Further inquiries can be directed to the corresponding author.

## Ethics Statement

All animal procedures were subject to institutional review by the Animal Welfare and Ethical Review Body and approved under UK Home Office Project License PPL 70/8169.

## Author Contributions

BY and CS designed and supervised the study. YW, ZZ, XZ, and HW carried out experiments. YW, ZZ, CS and BY analyzed the data and constructed the figures. RC supervised cell culture work. YW, BY, CS and NB wrote and revised the paper. All authors contributed to the article and approved the submitted version.

## Funding

This study was supported by the Leicester Kidney Care Appeal, the University Hospitals of Leicester NHS Trust Research and Innovation Department, the University of Leicester; project grants (81570677 and 81873622) from the National Natural Foundation of China; and also a project grant (JC2020036) from Nantong Science and Technology Foundation.

## Conflict of Interest

The authors declare that the research was conducted in the absence of any commercial or financial relationships that could be construed as a potential conflict of interest.

## Publisher’s Note

All claims expressed in this article are solely those of the authors and do not necessarily represent those of their affiliated organizations, or those of the publisher, the editors and the reviewers. Any product that may be evaluated in this article, or claim that may be made by its manufacturer, is not guaranteed or endorsed by the publisher.
