# Supplementary material for: Properdin Deficiency Impairs Phagocytosis and Enhances Injury at Kidney Repair Phase Post Ischemia–Reperfusion
Source: Front Immunol. 2021 Sep 6;12:697760. doi: 10.3389/fimmu.2021.697760 (PMC8450566; doi:10.3389/fimmu.2021.697760)
Supplement: Supplementary file 1 [file DataSheet_1.docx]

**Supplementary Material**


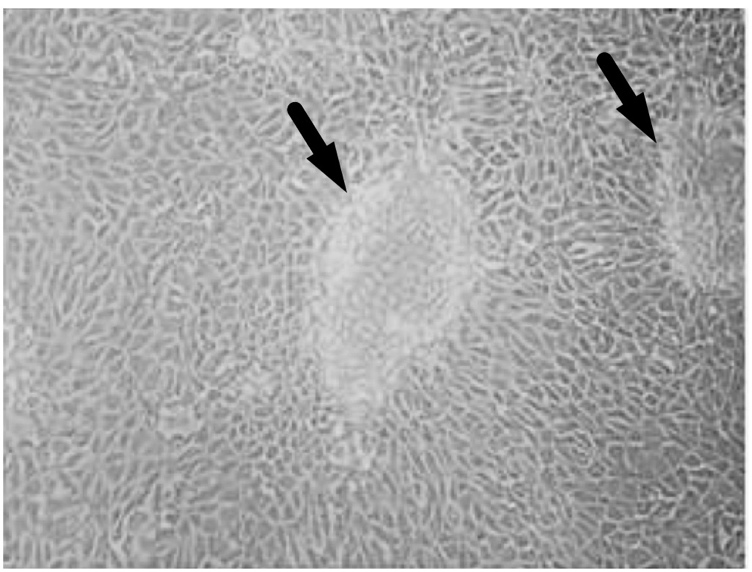


**Supplementary Figure 1**. **Dome formation in** **primary isolated TECs from mouse kidneys**. TECs were isolated from WT and P^KO^ C57BL/6 mice by size fraction and verified by various validated methods (30). A couple of domes (pointed by arrows) were formed and shown as examples in a micrograph from cultured WT TECs, but representing the phenomena of both WT and P^KO^ TECs. The formation of domes was due to an intact transcellular transport process, which is a characteristic of confluent TECs (31). Images were taken under 200× magnification.
